# New Binary Reptile Search Algorithms for Binary Optimization Problems

**DOI:** 10.3390/biomimetics10100653

**Published:** 2025-10-01

**Authors:** Broderick Crawford, Benjamín López Cortés, Felipe Cisternas-Caneo, José Manuel Gómez-Pulido, Rodrigo Olivares, Ricardo Soto, José Barrera-Garcia, Cristóbal Brante-Aguilera, Giovanni Giachetti

**Affiliations:** 1 Escuela de Ingeniería Informática, Pontificia Universidad Católica de Valparaíso, Avenida Brasil 2241, Valparaíso 2362807, Chile; benjamin.lopez.c@mail.pucv.cl (B.L.C.); felipe.cisternas.c@mail.pucv.cl (F.C.-C.); ricardo.soto@pucv.cl (R.S.); jose.barrera@pucv.cl (J.B.-G.); 2 Health Computing and Intelligent Systems Research Group (HCIS), Universidad de Alcalá, 28805 Alcalá de Henares, Madrid, Spain; jose.gomez@uah.es; 3 Department of Computer Science, Universidad de Alcalá, 28805 Alcalá de Henares, Madrid, Spain; 4 Ramón y Cajal Institute for Health Research (IRYCIS), 28034 Madrid, Spain; 5 Escuela de Ingeniería Informática, Universidad de Valparaíso, Valparaíso 2362905, Chile; rodrigo.olivares@uv.cl; 6 Escuela de Negocios y Economía, Pontificia Universidad Católica de Valparaíso, Amunátegui 1838, Viña del Mar 2580129, Chile; 7 Escuela de Ingeniería en Construcción y Transporte, Pontificia Universidad Católica de Valparaíso, Avenida Brasil 2147, Valparaíso 2362804, Chile; cristobal.brante@pucv.cl; 8 Facultad de Ingeniería, Universidad Andres Bello, Antonio Varas 880, Providencia, Santiago 7591538, Chile; giovanni.giachetti@unab.cl

**Keywords:** binarization process, transfer functions, binary rules, binary optimization problem, metaheuristics, reptile search algorithm, 68T20, 68W25, 90C27, 90C59, 68Q25

## Abstract

Binarizing continuous metaheuristics to solve challenging NP-hard binary optimization problems is a fundamental step in adapting continuous algorithms for discrete domains. Binary optimization problems, such as the Set Covering Problem and the 0–1 Knapsack Problem, demand tailored approaches to efficiently explore and exploit the solution space. The process of binarization often introduces complexities, as it requires balancing the transformation of continuous populations into binary solutions while preserving the algorithm’s capability to navigate the search space effectively. In this context, we explore the performance of the Reptile Search Algorithm (RSA), a continuous metaheuristic, applied to these two benchmark problems. To address the binary nature of the problems, a two-step binarization process is implemented, utilizing combinations of transfer functions with binarization rules. This framework enables the RSA to generate binary solutions while leveraging its inherent strengths in exploration and exploitation. Comparative experiments are conducted with Particle Swarm Optimization and the Grey Wolf Optimizer to benchmark the RSA’s performance under similar conditions. These experiments analyze critical factors such as fitness values, convergence behavior, and exploration–exploitation dynamics, providing insights into the effectiveness of different binarization approaches. The results demonstrate that the RSA achieves competitive performance across both problems, highlighting its flexibility and adaptability, which are attributed to its diverse movement equations. Notably, the Z4 transfer function consistently enhances performance for all algorithms, even when paired with less effective binarization rules. This indicates the potential of Z4 as a robust transfer function for binary optimization. The findings underscore the importance of selecting appropriate binarization strategies to maximize the performance of continuous metaheuristics in binary domains, paving the way for further advancements in hybrid optimization methodologies.

## 1. Introduction

In nearly every domain of human endeavor, resource scarcity poses a fundamental challenge. Whether the resources in question are physical—such as industrial materials, inventory, or energy—or abstract, like workforce availability, data, or time constraints, the demand for efficient allocation mechanisms remains ever-present. Optimization techniques play a crucial role in addressing these challenges by enabling us to maximize productivity and effectiveness in resource utilization.

Optimization problems can generally be categorized into two types: those involving discrete variables, where solutions are selected from a finite set (e.g., subsets of integers), and those with continuous variables, where solutions can take any real value. While continuous problems benefit from smoothness and mathematical properties like gradients to guide the search process, discrete problems are more challenging due to rugged search spaces. Despite this, discrete optimization has extensive applications in areas like load dispatch [1], dynamic vehicle routing [2], emergency humanitarian logistics [3], nurse scheduling [4], and vaccine distribution [5]. Exact methods, such as branch and cut [6] and branch and bound [7], offer robust solutions but often require significant computational resources for large-scale problems, making them impractical for time-sensitive applications.

Metaheuristic algorithms offer a compelling alternative. These algorithms are designed to balance exploration and exploitation within the solution space, making them well-suited for finding high-quality solutions efficiently, even for large and complex problems. Unlike exact methods, metaheuristics do not guarantee globally optimal solutions, but they provide approximate solutions of sufficient quality within acceptable computational timeframes. Furthermore, the no-free-lunch theorem mentioned in [8] emphasizes that no single optimization algorithm is universally superior, thereby fostering a continual stream of innovative metaheuristic designs tailored to specific problem characteristics. Recent examples of such algorithms include the Grey Wolf Optimizer [9], Honey Badger Algorithm [10], Eurasian Oystercatcher Optimizer [11], Sea-Horse Optimizer [12], and Fox Optimizer [13]. Classic algorithms such as Particle Swarm Optimization (PSO) [14] remain relevant and continue to perform well in many scenarios.

However, most of these metaheuristics are inherently designed for continuous domains. To adapt them for discrete optimization, a binarization process is required to transform the continuous solution space into a discrete one. This process enables continuous metaheuristics to tackle combinatorial problems by converting their continuous population representation into a format suitable for discrete problem spaces. Consequently, the effective design and evaluation of binarization schemes play a crucial role in determining the success of these algorithms in discrete applications.

In this work, we focus on evaluating the performance of the Reptile Search Algorithm (RSA) [15], a novel continuous metaheuristic, in solving binary combinatorial optimization problems. We explore its behavior under various binarization schemes and compare its performance to both contemporary and classic metaheuristics under identical conditions. By investigating the interplay between the binarization process and algorithmic performance, we aim to contribute valuable insights into the broader field of discrete metaheuristics.

The structure of this paper is as follows: Section 2 provides an overview of related work, including a detailed discussion of binarization techniques and the metaheuristics considered in this study. Section 3 describes the preliminaries, focusing on the mathematical foundations of the algorithms and the techniques used to adapt continuous metaheuristics to binary search spaces, such as transfer functions and binarization rules. Section 4 outlines the experimental design and methodological framework. Section 5 presents and analyzes the computational results obtained from the experiments. Finally, Section 6 concludes the paper by summarizing key findings and proposing directions for future research.

## 2. Related Work

Binarization strategies play a pivotal role in adapting continuous metaheuristics for solving binary optimization problems [16]. Numerous studies have explored innovative techniques to enhance the efficiency and effectiveness of these strategies [17]. Below, we summarize some of the key contributions in this domain.

One of the first proposals for the binarization of metaheuristics is the one by Kennedy and Eberhart in 1997 [18], where they propose a binarization for PSO. This binarization consists of binarizing the velocity of the particles, a key process for movement in the PSO search space.

Doe and Smith in [19] introduced a Binary Ant Lion Optimizer applied to the Knapsack problem, demonstrating how binarization can significantly improve the algorithm’s capability to address discrete optimization challenges. Similarly, Brown and White in [20] proposed a Binary Electric Eel Foraging Optimization Algorithm for the 0–1 Knapsack Problem, focusing on refining representation techniques and search mechanisms in the binary domain.

Xiong et al. in [21] developed a generalized method for binary optimization, offering convergence analysis and applications to various discrete problems. This study underscores how advancements in binarization methodologies contribute to enhancing algorithmic robustness and solution quality. Complementing this, Tian et al. in [22] presented a recommender system for metaheuristic algorithms based on deep recurrent neural networks. While not directly focused on binarization, the work indirectly supports binary optimization by tailoring algorithm selection to problem-specific characteristics.

A novel metaheuristic algorithm tailored for binary optimization was introduced by Von Dollen et al. in [23]. This study highlighted the importance of customized binarization techniques in achieving superior results for combinatorial optimization problems. Additionally, Jiang et al. in [24] proposed an enhanced Binary Grey Wolf Optimizer for dependent task scheduling in edge computing. This work illustrated how binarization strategies tailored to specific problem requirements can boost performance in real-world applications.

The work published by Thaher et al. in [25] explored the integration of evolutionary binarization techniques with Teaching–Learning-Based Optimization for feature selection. Their approach demonstrated the potential of hybrid strategies to balance exploration and exploitation effectively, addressing the challenges of binary optimization problems.

The authors in [26,27,28,29,30,31] explored advanced binarization strategies in metaheuristics for feature selection, emphasizing enhanced exploration–exploitation balance and efficient dimensionality reduction. Techniques like hybrid algorithms, novel transfer functions, and evolutionary approaches demonstrate significant improvements in classification accuracy and feature subset optimization. Collectively, these studies highlight the critical role of binarization in solving complex feature selection challenges across diverse datasets.

The advancement of machine learning has allowed the development of hybridizations with continuous metaheuristics. García et al. in [32] use unsupervised learning based on clustering for the binarization process of swarm intelligence algorithms. Crawford et al. in [33] propose the incorporation of reinforcement learning for the binarization process. Specifically, this learning technique aims to determine the best combinations of the transfer function and binarization rule that allow exploring and exploiting the search space.

Another interesting binarization approach is the quantum method, where the q-bit concept is used to perform the binarization process [34]. The authors in [35,36,37] highlight the use of this quantum approach to binarize continuous metaheuristics for the multidimensional knapsack problem, ordering problem, and feature selection problem.

These works collectively underline the importance of binarization in solving discrete optimization problems, demonstrating diverse approaches ranging from novel algorithms to advanced applications in specific domains. Our work, motivated by the no-free-lunch theorem [38,39], builds upon these foundational studies by exploring a two-step binarization process within the RSA, comparing its effectiveness against established methods.

## 3. Preliminaries

In this section, main topics will be addressed. First, metaheuristic algorithms will be discussed, including a detailed explanation of the Reptile Search Algorithm and the metaheuristics used for comparison: the Grey Wolf Optimizer and Particle Swarm Optimization. Then, the two-step technique will be introduced as a method for applying continuous metaheuristics to binary problems.

### 3.1. Reptile Search Algorithm

The Reptile Search Algorithm is a metaheuristic proposed by Laith Abualigah et al. [15], inspired by the encircling and hunting behaviors observed in crocodiles, along with their social dynamics. This algorithm balances exploration and exploitation of the search space using four distinct equations, alternating through iterations. Two key parameters are used to control the exploration accuracy, defined as the variation between candidate solutions.

The exploration phase, referred to as the Encircling Phase, encompasses two movement equations inspired by the high-walking and belly-walking behaviors of crocodiles while encircling their prey. These movements create disturbances that prevent the crocodiles from approaching their target directly, leading this phase to prioritize wide search space exploration.

In contrast, the exploitation phase, known as the *Hunting Phase*, also includes two movement equations derived from crocodile hunting strategies: hunting coordination and hunting cooperation. These strategies allow crocodiles to intensify their efforts and effectively approach their target. Consequently, this phase emphasizes discovering near-optimal solutions through focused exploitation of the search space.

The algorithm alternates between exploration and exploitation phases based on the number of iterations. Each equation operates during a specific quarter of the total iterations, transitioning from exploration to exploitation at the midpoint of the search process. The equations for exploration (1) and exploitation (2) are defined as follows:

- Encircling Phase: The equations for high-walking and belly-walking are
  (1) $$x_{\left(i,j\right)}\left(t+1\right)=\left\{\begin{array}{c} Best_{j}\left(t\right)\times -\eta_{\left(i,j\right)}\left(t\right)\times \beta -R_{\left(i,j\right)}\left(t\right)\times rand,\;\mathrm{if}\;t\leq \frac{T}{4}\\ Best_{j}\left(t\right)\times x_{\left(r_{1},j\right)}\times ES\left(t\right)\times rand,\;\mathrm{if}\;\frac{T}{4}<t\leq \frac{T}{2}. \end{array}\right.$$
- Hunting Phase: The equations for hunting coordination and hunting cooperation are
  (2) $$x_{\left(i,j\right)}\left(t+1\right)=\left\{\begin{array}{cc} Best_{j}\left(t\right)\times P_{\left(i,j\right)}\left(t\right)\times rand, & \frac{T}{2}<t\leq \frac{3T}{4},\\ Best_{j}\left(t\right)-\eta_{\left(i,j\right)}\left(t\right)\times \epsilon -R_{\left(i,j\right)}\left(t\right)\times rand, & \frac{3T}{4}<t\leq T. \end{array}\right.$$
  In these formulas, $Best_{j}\left(t\right)$ represents the *j*th position in the best solution found so far, while rand is a random number between 0 and 1. The variable *t* denotes the current iteration, and *T* is the total number of iterations. The parameter β takes a value between 0 and 1. The variables $r_{1}$ and $r_{2}$ are random integers between 1 and *N*, where *N* is the number of candidate solutions. Additionally, $\eta_{\left(i,j\right)}$, $R_{\left(i,j\right)}$, ES(t), and $P_{\left(i,j\right)}$ are computed as follows:
  (3) $$\eta_{\left(i,j\right)}=Best_{l}\left(t\right)\times P_{\left(i,j\right)},$$
  (4) $$R_{\left(i,j\right)}=\frac{Best_{j}\left(t\right)-x_{\left(r_{2},j\right)}}{Best_{j}\left(t\right)+\epsilon },$$
  (5) $$ES\left(t\right)=2\times r_{3}\times \left(1-\frac{t}{T}\right),$$
  (6) $$P_{\left(i,j\right)}=\alpha +\frac{x_{\left(i,j\right)}-M\left(x_{i}\right)}{Best_{j}\left(t\right)\times \left(UB_{\left(j\right)}-LB_{\left(j\right)}\right)+\epsilon }.$$
  where ϵ is a small constant to prevent division by zero, $r_{3}$ is a random integer between −1 and 1, and $UB_{\left(j\right)}$ and $LB_{\left(j\right)}$ represent the upper and lower bounds of the *j*th position, respectively. The parameter α takes a value between 0 and 1. Additionally, $M(x_{i})$, the average position of the *i*th solution, is calculated as
  (7) $$M\left(x_{i}\right)=\frac{1}{N}\sum_{j=1}^{n}x_{\left(i,j\right)}.$$

This study evaluates the Reptile Search Algorithm’s performance, including its exploration and exploitation dynamics and the diversity of solutions it generates. Two other metaheuristics will also be tested for comparative purposes, ensuring a comprehensive analysis.

### 3.2. Particle Swarm Optimization

Particle Swarm Optimization is a foundational metaheuristic algorithm introduced by Kennedy and Eberhart in [14]. It draws inspiration from sociobiological theories, particularly the observation that groups of animals, such as schools of fish or flocks of birds, benefit from sharing discoveries and leveraging the prior experiences of individual members during their collective search for resources [40]. This behavioral analogy forms the core principle of PSO, which enables optimization by mimicking the way these groups collaborate and adapt in dynamic environments.

One of the primary advantages of PSO is its simplicity. The algorithm requires only basic mathematical operations and is computationally efficient, both in terms of memory usage and processing speed. This makes PSO highly accessible and applicable to a wide variety of optimization problems, ranging from engineering design to machine learning and beyond.

The PSO algorithm operates by maintaining a swarm of *P* particles, where each particle represents a candidate solution in the search space. Each particle is characterized by its position and velocity, which are iteratively updated based on its own experience and the experiences of others in the swarm. The update rules for position and velocity are as follows:

(8) $$X_{i}\left(t+1\right)=X_{i}\left(t\right)+V_{i}\left(t+1\right),$$

where $X_{i}\left(t\right)$ denotes the position of the *i*th particle at iteration *t*, and $V_{i}\left(t+1\right)$ represents the velocity of the *i*th particle at iteration t+1. The velocity update equation is given by

(9) $$V_{\left(i,j\right)}\left(t+1\right)=w\times V_{\left(i,j\right)}\left(t\right)+c_{1}\times r_{1(i,j)}\times \left(pBest_{\left(i,j\right)}-X_{\left(i,j\right)}\left(t\right)\right)+c_{2}\times r_{2(i,j)}\times \left(gBest_{j}-X_{\left(i,j\right)}\left(t\right)\right),$$

where $c_{1}$ and $c_{2}$ are acceleration coefficients that determine the influence of the personal best and global best positions, respectively; $r_{1(i,j)}$ and $r_{2(i,j)}$ are random numbers uniformly distributed between 0 and 1, introducing stochasticity into the search process; $pBest_{\left(i,j\right)}$ is the personal best position found by the *i*th particle in the *j*th dimension; and $gBest_{j}$ is the global best position found by the entire swarm in the *j*th dimension. *w* is the inertia weight, which balances exploration and exploitation by controlling the influence of the previous speed, which is calculated as indicated in Equation (10).

(10) $$w=w_{max}-iter\cdot \frac{w_{max}-w_{min}}{maxIter},$$

where $w_{max}$ and $w_{min}$ are the limits of the inertia weight, which is defined as 0.9 and 0.1, respectively; iter indicates the current iteration and maxIter indicates the maximum iterations of the algorithm, therefore, the inertia weight (*w*) starts at 0.9 and gradually decreases to 0.1 as the iterations progress.

The algorithm proceeds by iteratively updating the positions and velocities of all particles in the swarm. Each particle is influenced by its own best position (pBest) and the swarm’s best-known position (gBest), promoting both individual and collective learning. The inertia weight (*w*) plays a critical role in balancing exploration and exploitation. Higher values of *w* encourage exploration of the search space, while lower values promote convergence by focusing on exploitation of the known promising regions.

PSO’s simplicity and flexibility have contributed to its widespread adoption in solving both continuous and discrete optimization problems. However, it is important to note that the algorithm is highly sensitive to parameter tuning. Improper selection of *w*, $c_{1}$, and $c_{2}$ can lead to premature convergence or slow exploration. Various adaptive and hybrid approaches have been proposed in the literature to address these challenges and enhance PSO’s performance across diverse applications.

### 3.3. Grey Wolf Optimizer

The Grey Wolf Optimizer was proposed by Mirjalili et al. in [9] and is inspired by the social hierarchy and hunting behavior of grey wolves. Wolves in a pack are organized into a social structure comprising four roles: alpha (α), beta (β), delta (δ), and omega (ω). The alpha wolf is the leader and the most dominant individual, responsible for making decisions and guiding the pack. The beta wolf serves as an advisor and enforcer of the alpha’s commands while maintaining discipline within the pack. The delta wolves take on roles such as scouts, sentinels, or hunters, supporting the leadership. Finally, the omega wolves are the lowest-ranking members, often subordinate but playing supportive roles such as caretakers.

In the context of optimization, the GWO models this hierarchy by assigning the best candidate solution as the alpha (α), the second best as the beta (β), and the third best as the delta (δ). All other solutions are considered omegas (ω), which follow the guidance of the three top-ranked wolves during the optimization process. This dynamic allows the algorithm to simulate the hunting behavior of wolves, leveraging both exploration and exploitation in the search space.

The position of each wolf in the search space is updated using the following equations:

(11) $$\vec{X}\left(t+1\right)={\vec{X}}_{p}\left(t\right)-\vec{A}\cdot \vec{D}$$

(12) $$\vec{D}=\left|\vec{C}\cdot {\vec{X}}_{p}\left(t\right)-\vec{X}\left(t\right)\right|$$

Here, *t* is the current iteration, ${\vec{X}}_{p}$ represents the position vector of the prey (optimal solution), and $\vec{X}$ is the position vector of a grey wolf. The vectors $\vec{A}$ and $\vec{C}$ are coefficient vectors, defined as:

(13) $$\vec{A}=2\vec{a}\cdot {\vec{r}}_{1}-\vec{a}$$

(14) $$\vec{C}=2\cdot {\vec{r}}_{2}$$

The components of $\vec{a}$ linearly decrease from 2 to 0 over the iterations, ${\vec{r}}_{1}$ and ${\vec{r}}_{2}$ are random vectors uniformly distributed in [0, 1]. The parameter $\vec{A}$ manages the balance between exploration (searching new regions) and exploitation (intensifying search around known good solutions), while $\vec{C}$ introduces randomness to help the algorithm escape local optima.

To simulate the hunting process in an unknown search space, the positions of wolves are guided by the best solutions obtained so far. The following equations model this behavior:

(15) $$\vec{D_{\alpha }}=\left|\vec{C_{1}}\cdot \vec{X_{\alpha }}-\vec{X}\right|,\;\vec{D_{\beta }}=\left|\vec{C_{2}}\cdot \vec{X_{\beta }}-\vec{X}\right|,\;\vec{D_{\delta }}=\left|\vec{C_{3}}\cdot \vec{X_{\delta }}-\vec{X}\right|$$

(16) $${\vec{X}}_{1}=\vec{X_{\alpha }}-{\vec{A}}_{1}\cdot \vec{D_{\alpha }},\;{\vec{X}}_{2}=\vec{X_{\beta }}-{\vec{A}}_{2}\cdot \vec{D_{\beta }},\;{\vec{X}}_{3}=\vec{X_{\delta }}-{\vec{A}}_{3}\cdot \vec{D_{\delta }}$$

(17) $$\vec{X}\left(t+1\right)=\frac{{\vec{X}}_{1}+{\vec{X}}_{2}+{\vec{X}}_{3}}{3}$$

Here, ${\vec{X}}_{\alpha }$, ${\vec{X}}_{\beta }$, and ${\vec{X}}_{\delta }$ are the positions of the alpha, beta, and delta wolves, respectively. $\vec{X}$ is the current position of the wolf, and $\vec{X}\left(t+1\right)$ is the updated position. By averaging the contributions of the three leading wolves, the algorithm ensures a balanced search behavior that progressively converges to the optimal solution.

The Grey Wolf Optimizer stands out due to its simplicity, lack of extensive hyperparameter tuning, and ability to achieve competitive results in diverse optimization scenarios. Its biologically inspired structure provides a flexible framework for solving both continuous and discrete optimization problems when adapted appropriately.

### 3.4. Two-Step Technique

To adapt continuous metaheuristics for discrete optimization problems, a binarization framework is applied. This framework consists of two steps: first, transfer functions convert continuous values into probabilities; second, binarization rules use these probabilities to produce binary solutions [16]. Additionally, population diversity plays a critical role in evaluating the balance between exploration and exploitation, which is essential for the algorithm’s efficiency. The following subsections detail transfer functions, binarization rules, and the role of diversity in guiding optimization processes.

#### 3.4.1. Transfer Functions

The transfer function provides a probabilistic mapping for continuous population values, offering a computationally inexpensive approach to binarization. Numerous transfer functions exist, with no universally superior option, as their performance depends on the specific problem. Table 1 presents S-shaped, V-shaped, X-shaped, and Z-shaped transfer functions [41,42,43,44]. These functions calculate probabilities based on the continuous values of the dimensions of candidate solutions.

#### 3.4.2. Binarization Rules

Once probabilities are calculated using transfer functions, binarization rules convert these probabilities into binary values. Table 2 lists commonly used binarization rules [16,17]. The choice of rule impacts performance and must align with the problem’s requirements to achieve optimal results.

#### 3.4.3. Diversity

Population diversity provides insights into the exploration and exploitation dynamics of an algorithm. It measures the variation among candidate solutions and helps identify whether the algorithm is exploring new areas or exploiting known promising regions. Hussain et al. in [45] formalized diversity as follows:

(18) $$Div=\frac{1}{l\cdot n}\sum_{d=1}^{l}\sum_{i=1}^{n}\left|x^{-d}-x_{i}^{d}\right|,$$

where Div is the diversity value, $x^{-d}$ is the mean of all individuals in the *d*th dimension, $x_{i}^{d}$ is the value of the *i*th individual in the *d*th dimension, *n* is the population size, and *l* is the dimension size.

Using diversity, the exploration percentage (XPL%) and exploitation percentage (XPT%) are calculated as proposed by Morales–Castañeda et al. in [46]:

(19) $$XPL\%=\frac{Div}{Div_{max}}\cdot 100,$$

(20) $$XPT\%=\frac{|Div-Div_{max}|}{Div_{max}}\cdot 100,$$

where $Div_{max}$ is the maximum diversity value observed during the optimization process. This information will be used to analyze the behavior of the algorithms during the optimization process in terms of exploration-exploitation.

## 4. Experimental Setup

This section describes the experimental setup designed to evaluate the performance of three continuous metaheuristics—the Reptile Search Algorithm, the Grey Wolf Optimizer, and Particle Swarm Optimization—on two classical combinatorial optimization problems: the Set Covering Problem (SCP) and the Knapsack Problem (KP). The choice of Particle Swarm Optimization and Grey Wolf Optimizer is based on a recent literature review indicating the relevance of both in the field [47].

Given the combinatorial nature of these problems, the continuous populations generated by the metaheuristics must be converted into binary solutions. This is achieved through a two-step binarization process, which first applies transfer functions to transform continuous values into probabilities, followed by binarization rules that map these probabilities into binary solutions. This methodology enables continuous metaheuristics to effectively address discrete optimization problems, as discussed in previous sections.

To comprehensively evaluate the performance of the metaheuristics under diverse scenarios, the experiments combine two transfer functions (X-shaped and Z-shaped) with three binarization rules (Standard, Complement, and Elitist). Each metaheuristic was tested across all possible configurations of these transfer functions and binarization rules. For example, the Reptile Search Algorithm paired with the Z-shaped transfer function and the Elitist binarization rule emphasized intensification, focusing on exploiting promising regions of the search space. In contrast, Particle Swarm Optimization combined with the X-shaped transfer function and the Complement binarization rule aimed to enhance exploration by introducing greater variability in the binarization process. These setups ensured a thorough exploration of the algorithms’ capabilities in balancing exploration and exploitation.

### 4.1. Set Covering Problem

The SCP is a well-known NP-hard combinatorial optimization problem with applications in resource allocation, logistics, and scheduling. The SCP involves finding a subset of sets that covers all elements in a universal set while minimizing the total cost. The problem can be formulated mathematically as follows:

(21) $$\mathrm{minimize}\;Z=\sum_{j=1}^{n}c_{j}x_{j},$$

subject to:

(22) $$\sum_{j=1}^{n}a_{ij}x_{j}\geq 1,\;\forall i\in \left\{1,2,\ldots ,m\right\},$$

(23) $$x_{j}\in \left\{0,1\right\},\;\forall j\in \left\{1,2,\ldots ,n\right\},$$

where $A=[a_{ij}]$ is an m×n binary matrix, with $a_{ij}=1$ if set *j* covers element *i*, and $a_{ij}=0$ otherwise. Each column *j* has an associated cost $c_{j}$, and $x_{j}$ is a binary decision variable indicating whether set *j* is included in the solution.

In this study, instances from Beasley’s OR-Library are used [48]. Specifically, the first instances of sets 4 to NRF are selected to evaluate algorithm performance across diverse problem sizes and complexities.

### 4.2. 0–1 Knapsack Problem

The 0–1 Knapsack Problem is another classic NP-hard problem that involves selecting a subset of items to maximize total value while ensuring that the total weight does not exceed the knapsack’s capacity. The problem is formulated as follows:

(24) $$\mathrm{maximize}\;Z=\sum_{i=1}^{n}v_{i}x_{i},$$

subject to

(25) $$\sum_{i=1}^{n}w_{i}x_{i}\leq C,$$

(26) $$x_{i}\in \left\{0,1\right\},\;\forall i\in \left\{1,2,\ldots ,n\right\},$$

where $x_{i}$ is a binary decision variable indicating whether item *i* is included in the knapsack ($x_{i}=1$) or not ($x_{i}=0$). $v_{i}$ and $w_{i}$ represent the value and weight of item *i*, respectively, and *C* is the maximum capacity of the knapsack.

For this work, instances from Pisinger’s dataset are employed [49]. These include instances with 500, 1000, 2000, and 5000 items across uncorrelated, weakly correlated, and strongly correlated datasets, allowing for a robust comparison of algorithm performance under varying conditions.

### 4.3. Experimental Details

The algorithms were implemented in Python 3.11 and executed on a private server equipped with an AMD Ryzen 7 5800X processor (Advanced Micro Devices, Silicon Valley, CA, USA) and 32 GB of DDR4 RAM. Each metaheuristic algorithm employed a population of 20 individuals and a maximum of 500 iterations across all experiments. To ensure statistical reliability, 31 independent runs were performed for each instance. Table 3 shows the parameters of the three metaheuristics used in this work.

The evaluation focuses on three key aspects: (1) convergence plots to illustrate optimization progress, (2) analysis of exploration and exploitation behaviors to assess balance, and (3) statistical tests comparing performance across configurations. This comprehensive evaluation provides insights into the strengths and weaknesses of each algorithm in addressing complex combinatorial problems.

## 5. Computational Results

This section presents an analysis of the results obtained using the three metaheuristics discussed earlier. Table 4, Table 5, Table 6, Table 7, Table 8 and Table 9 display the best fitness achieved among all 31 experiments, the average value of all fitnesses across the 31 experiments, and the relative percentage deviation (RPD), grouped by each SCP or KP instance. Each row in these tables represents a specific combination of metaheuristic, transfer function, and binarization rule.

The calculation of the RPD varies depending on the optimization problem. For minimization optimization problems such as the Set Covering Problem, the RPD is calculated as indicated in Equation (27). On the other hand, for maximization optimization problems such as the Knapsack Problem, the RPD is calculated as indicated in Equation (28). Both measures provide a relative deviation from the optimal solution, indicating the performance of each configuration.

(27) $$RPD_{\mathrm{minimization}}=\frac{100\cdot (Opt-Best)}{Opt}$$

(28) $$RPD_{\mathrm{maximization}}=\frac{100\cdot (Best-Opt)}{Opt}$$

where Opt corresponds to the optimum of the instance and Best corresponds to the best value obtained for the experiment.

For the Set Covering Problem results, as summarized in Table 4, Table 5 and Table 6, all algorithms demonstrated competitive performance when paired with appropriate combinations of transfer functions and binarization rules. A notable observation is that the Elitist binarization rule consistently delivered the best results across most scenarios, particularly when used with both the Z4 and X4 transfer functions. In contrast, the Standard and Complement binarization rules showed mixed performance. When paired with the Z4 function, these rules provided competitive results, occasionally rivaling the performance of the Elitist rule. However, when combined with the X4 function, the algorithms’ performance significantly declined. The worst performance was observed with the X4–Complement pairing, where the minimum RPD obtained was 752, recorded on the SCP41 instance using RSA.

Overall, the RSA emerged as the top performer. The RSA achieved the optimal solution in seven combinations, spanning instances such as scpnre1 (Z4–Standard, Z4–Complement, Z4–Elitist, and X4–Elitist) and scpnrf1 (Z4–Standard, Z4–Complement, and X4–Elitist). PSO followed, achieving the optimum in four combinations, including scpnre1 (Z4–Elitist and X4–Elitist) and scpnrf1 (Z4–Elitist and X4–Elitist). The GWO achieved the optimum in only two combinations (scpnrf1 with Z4–Standard and X4–Elitist). Even under the less effective binarization schemes (X4–Standard and X4–Complement), the RSA mitigated performance loss better than PSO and the GWO, consistently achieving lower RPD values.

Notably, there are only four cases where a metaheuristic achieved a lower RPD than the RSA, all of which involve PSO. Three of these cases are observed under the X4–Elitist scheme for the SCP51, SCPC1, and SCPD1 instances. The fourth case is under the Z4–Elitist scheme for the SCPNRF1 instance. It is worth emphasizing that, in all these instances, RSA’s fitness value was just one unit larger than PSO’s.

There are eleven instances where RSA matched the RPD achieved by other algorithms. With a total of 162 possible combinations (12 metaheuristic and binarization schemes across 9 SCP instances), the RSA either equaled or outperformed the other algorithms in 158 cases. Specifically, RSA matched performance in 11 cases, fell slightly behind PSO in 4 cases, and outperformed PSO and the GWO in the remaining 147 cases.

In the case of the 0–1 Knapsack Problem results, as summarized in Table 7, Table 8 and Table 9, all algorithms demonstrated competitive performance when paired with appropriate combinations of transfer functions and binarization rules. Among the binarization rules, the Standard rule emerged as the most effective, delivering consistent results when paired with both Z4 and X4 transfer functions in the PSO and GWO experiments. However, the Reptile Search Algorithm (RSA) showed a notable advantage when paired with the Z4 transfer function and the Standard binarization rule, significantly outperforming the other configurations.

Contrary to the trends observed in the Set Covering Problem, the Elitist binarization rule performed poorly in the KP experiments. This highlights the problem-dependent nature of binarization rules and transfer functions, as strategies that excel in one context may underperform in another.

The results across KP instances were generally more consistent, with performance values staying closer to the optimum. The worst-case RPD was 20%, observed with the GWO algorithm using the X4–Complement binarization scheme. Despite this, none of the algorithms delivered poor results overall. The average RPD across all experiments was 7.71%, with a standard deviation of 4.96%. These values are significantly better compared to the SCP results, which had a much higher average RPD of 32.63% and a standard deviation of 50.34%. This disparity can likely be attributed to the SCP experiments’ susceptibility to extreme values caused by the combination of the X4 transfer function with the Standard and Complement binarization rules across all algorithms.

The RSA consistently outperformed PSO and the GWO in the KP instances, achieving the only zero-valued RPD in the experiments (Instance knapPI_3_5000_1000_1 with the Z4–Standard binarization scheme). While the RSA’s performance was generally close to that of PSO and the GWO, the Z4–Standard binarization scheme provided a significant boost, achieving RPD values below 1% across all KP instances. This result underscores the importance of carefully selecting the binarization scheme, as it can unlock the full potential of an algorithm. The RSA paired with Z4-Standard consistently excelled, while other binarization schemes did not fully leverage RSA’s capabilities.

It is worth noting that RSA was surpassed in only one instance (knapPI_2_500_1000_1), where both PSO and GWO marginally outperformed RSA using the X4–Standard binarization scheme. However, even in this case, the performance gap was minimal, reaffirming the RSA’s overall robustness across the KP instances.

### 5.1. Convergence Analysis

**Figure 1:**
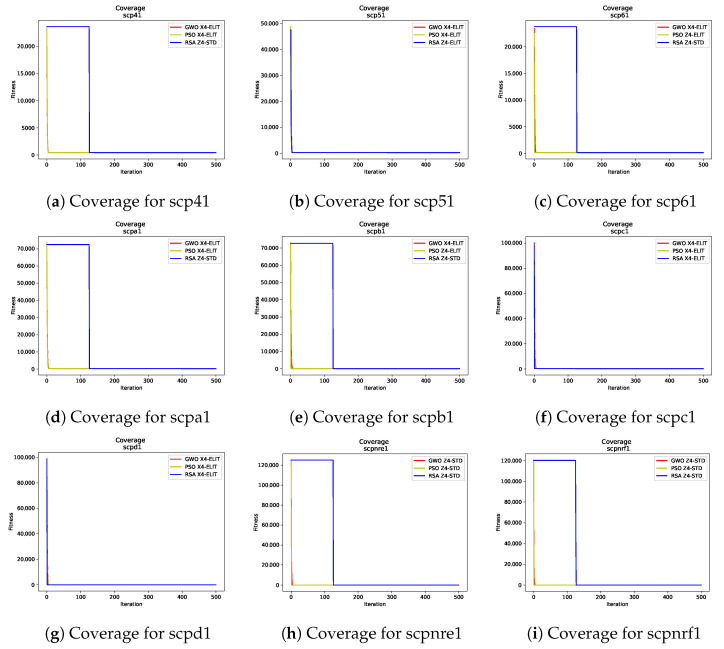
Convergence graphs (iterations vs. fitness) for all SCP instances and metaheuristics. Each subfigure illustrates the convergence behavior of the best runs for the corresponding instance, highlighting the impact of binarization schemes and transfer functions.

**Figure 2:**
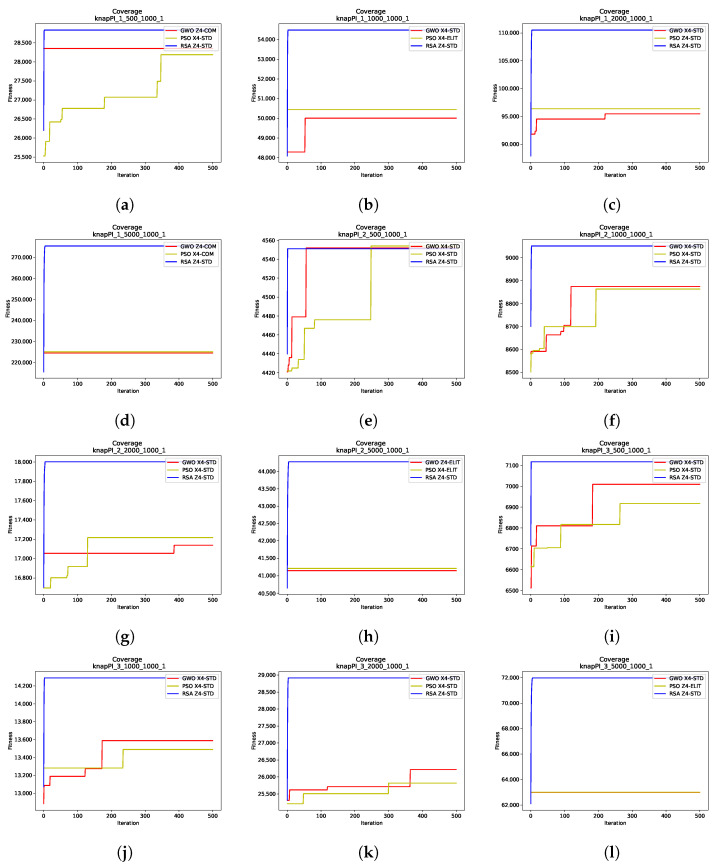
Convergence graphs (iterations vs. fitness) for all 0–1 Knapsack Problem instances. Each subfigure illustrates the convergence behavior of the best runs for the corresponding instance. (**a**) Coverage of knapPI_1_500_1000_1. (**b**) Coverage of knapPI_1_1000_1000_1. (**c**) Coverage of knapPI_1_2000_1000_1. (**d**) Coverage of knapPI_1_5000_1000_1. (**e**) Coverage of knapPI_2_500_1000_1. (**f**) Coverage of knapPI_2_1000_1000_1. (**g**) Coverage of knapPI_2_2000_1000_1. (**h**) Coverage of knapPI_2_5000_1000_1. (**i**) Coverage of knapPI_3_500_1000_1. (**j**) Coverage of knapPI_3_1000_1000_1. (**k**) Coverage of knapPI_3_2000_1000_1. (**l**) Coverage of knapPI_3_5000_1000_1.

To analyze the convergence behavior of the nine SCP instances and the twelve 0–1 KP instances across the three algorithms, Figure 1 and Figure 2 display the convergence curves for the best runs of each algorithm in the respective instances. These figures highlight the binarization schemes employed by the metaheuristics, providing context for the results previously analyzed.

For the Set Covering Problem, the convergence curves in Figure 1 reveal distinct patterns among the algorithms. PSO and GWO demonstrate rapid convergence, significantly reducing the fitness value within the first few iterations. In contrast, the RSA exhibits a delayed reduction in fitness during the first quarter of iterations, with the exception of Figure 1b,f. This delay can be attributed to the RSA High Walking, which appears less effective in the initial exploration phase for this specific problem. However, after the first quarter of iterations, the RSA’s other movement equations contribute significantly to its performance, resulting in competitive fitness values.

It is noteworthy that all algorithms begin with high fitness values, leading to a sharp initial decline in all curves. For RSA, this sharp decline occurs slightly later due to the transition to its second phase, which appears better suited for SCP resolution. The results indicate that the Elite binarization rule dominates among the best-performing configurations, with the X4 transfer function frequently appearing in Figure 1a,b,d,f,g. However, for the largest SCP instances (Figure 1h,i), all algorithms consistently rely on the Z4–Standard binarization scheme.

For the 0–1 Knapsack Problem, the convergence curves in Figure 2 present a markedly different pattern. Unlike in the SCP, the RSA demonstrates a sharp initial reduction in fitness, quickly achieving high fitness values and maintaining relative stability throughout the iterations. In comparison, both PSO and the GWO show gradual improvements, incrementally reaching stable fitness values. This gradual behavior is particularly evident in smaller KP instances, as seen in Figure 2a,e–g,i–k. In larger instances, such as those in Figure 2d,h,l, both PSO and the GWO struggle to surpass the initial solution fitness throughout the entire process.

The results highlight that the RSA achieves its best performance using the Z4–Standard binarization scheme. PSO and the GWO also deliver their best results with the Standard binarization rule, although some cases involving the Complement and Elitist rules (Figure 2d,h,k) show competitive results. These instances, however, likely reflect the challenges posed by the larger KP instances, where PSO and the GWO struggle to improve upon their initial solutions.

Finally, we can state that the convergence analysis underscores the importance of selecting appropriate binarization schemes and transfer functions for each problem. While the RSA demonstrates slower initial progress in the SCP, it compensates with strong performance in later stages, particularly when paired with the Z4–Standard binarization scheme. In the KP, the RSA’s rapid initial convergence and stability further highlight its robustness compared to PSO and the GWO. These findings reaffirm the critical role of algorithmic configurations in optimization problems.

### 5.2. Exploration and Exploitation Analysis

**Figure 3:**
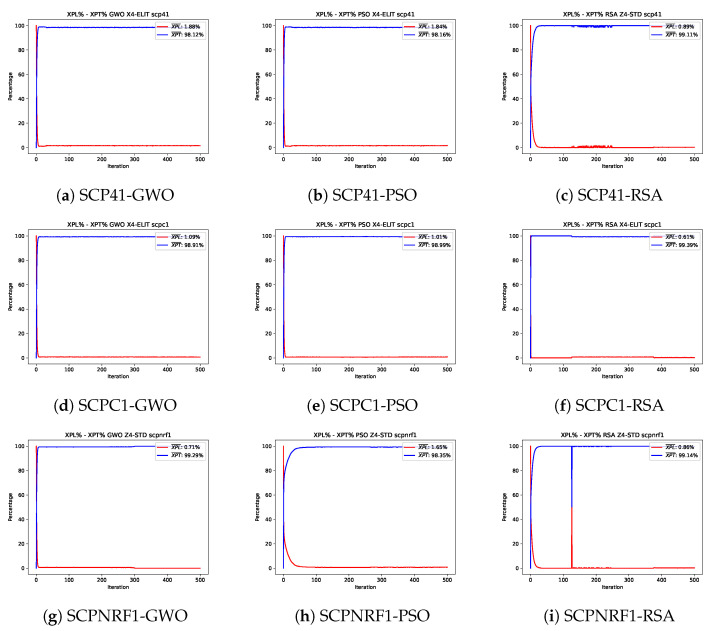
Exploration–exploitation graphs of the best executions obtained for the scp41, scpc1, and scpnrf1 instances.

**Figure 4:**
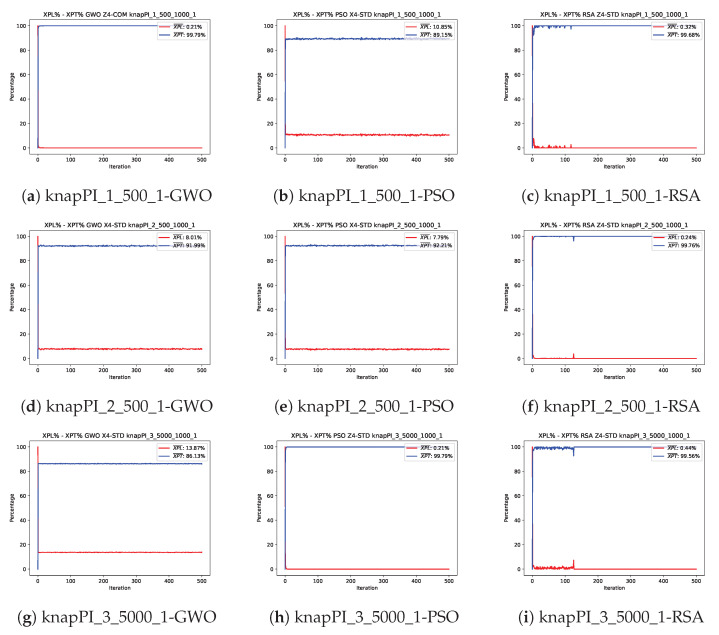
Exploration–exploitation graphs of the best executions obtained for the knapPI_1_500_1, knapPI_2_500_1, and knapPI_3_5000_1 instances.

This section examines the exploration and exploitation behavior exhibited by the three selected algorithms. Figure 3 and Figure 4 depict the analysis for three selected instances of the Set Covering Problem (SCP) and the 0–1 Knapsack Problem (KP), respectively. These figures illustrate the behavior of the algorithms during their best runs.

For the SCP, the exploration and exploitation curves are relatively stable for all algorithms, with the exception of the Reptile Search Algorithm, which displays notable changes throughout the iterations. The abrupt shifts observed in the convergence curves of PSO and GWO for the scp41 and scpc1 instances (Figure 3e). These show that the initially high exploration value is quickly replaced by a high exploitation value within the first few iterations. This behavior persists until the end of the process, likely because these two algorithms reached a local optimum early in the execution.

The RSA follows a similar trend to PSO and the GWO, rapidly transitioning to high exploitation values shortly after the execution begins. However, it is worth noting that the RSA exhibits spikes in exploration behavior between the second and third quarters of the iterations. This aligns with the convergence graphs, where the RSA does not significantly reduce the fitness value of the population during the first quarter of the iterations. After this period, as a new movement equation is applied, the fitness value decreases sharply, suggesting that the subsequent equations are better suited for exploring the SCP search space.

In the case of the KP, the exploration and exploitation curves shown in Figure 4 exhibit different patterns compared to those observed for the SCP. The curves for the GWO (Figure 4a,d) and PSO (Figure 4e,h) remain in a nearly constant state of approximately 90% exploitation and 10% exploration. This behavior is likely due to the search capabilities of these algorithms within the solution space for this specific problem. In many instances, particularly the larger ones, both algorithms fail to identify higher-quality solutions throughout the process.

In contrast, the RSA exhibits a behavior similar to its performance in the SCP, with an abrupt shift towards higher exploitation percentages due to the rapid identification of high-quality solutions. However, all RSA graphs across the studied instances show spikes in exploration during the first quarter of the iterations, similar to the spikes observed in the SCP curves between the second and third quarters of the iterations. This suggests that the RSA’s first equation is better suited for the KP, enabling a rapid increase in fitness value within the initial iterations. Conversely, in the SCP, the algorithm requires the subsequent equations to achieve better fitness-valued solutions.

### 5.3. Statistical Test

To rigorously evaluate the significance of these observations, the Wilcoxon–Mann–Whitney test was applied to the obtained results. This is a non-parametric statistical test [50], particularly suitable for data that is not normally distributed and for comparing independent samples. These conditions align with the characteristics of our experimental setup, where the data from each experiment does not follow a normal distribution, and the runs are performed independently.

For the statistical analysis, 31 runs were conducted for each combination of algorithm and binarization scheme. The hypotheses tested for the SCP and KP cases were formulated to reflect the specific optimization goals of each problem:

Set Covering Problem

- $H_{0}$: Algorithm A produces results that are not better than Algorithm B.
- $H_{1}$: Algorithm A produces results that are better than Algorithm B.

0–1 Knapsack Problem

- $H_{0}$: Algorithm A produces results that are not worse than Algorithm B.
- $H_{1}$: Algorithm A produces results that are worse than Algorithm B.

The null hypothesis ($H_{0}$) represents the assumption of no significant difference in performance between the algorithms under comparison. If the resulting *p*-value is less than 0.05, $H_{0}$ is rejected, indicating that Algorithm A either outperforms or underperforms Algorithm B, depending on the specific hypothesis tested.

To ensure a comprehensive analysis, each individual problem instance underwent its own statistical test. With nine SCP instances and 12 KP instances analyzed across three algorithms, a total of 63 statistical tests were conducted. Each test examined the influence of the binarization scheme and transfer function on the algorithm’s performance for a particular problem instance.

Given the volume of statistical tests performed, the results are summarized for clarity. Instead of presenting 63 individual tables, the outcomes were aggregated into six summary tables—one for each algorithm across both SCP and KP cases. These tables count the number of times a specific binarization scheme achieved a *p*-value below 0.05 in the tests. This aggregation provides a concise overview of the statistical significance of each binarization scheme’s contribution to the algorithms’ performance.

The statistical analysis revealed distinct patterns in algorithm performance.

1. Set Covering Problem: The Elitist binarization rule frequently achieved the most significant results, particularly when paired with the Z4 and X4 transfer functions. This observation aligns with the findings, where these combinations consistently delivered the best solutions across SCP instances.
2. The 0–1 Knapsack Problem: The Standard binarization rule paired with the Z4 transfer function demonstrated superior performance, achieving statistically significant results in most KP instances. This reflects the algorithm’s ability to effectively balance exploration and exploitation under this configuration.
3. Across both problems, the Reptile Search Algorithm emerged as the most consistent performer, showing a higher count of statistically significant outcomes compared to PSO and the GWO. This underscores the robustness of RSA, particularly when paired with the Z4–Standard binarization scheme.

The statistical tests reinforce the importance of selecting appropriate binarization schemes and transfer functions tailored to the problem at hand. While some configurations yielded universally strong results, others were more problem-specific. By systematically analyzing the *p*-values, we were able to identify the configurations that consistently enhanced algorithm performance. These findings provide valuable insights into the design and tuning of metaheuristic algorithms for combinatorial optimization problems.

Table 10 provides a comprehensive analysis of the Wilcoxon–Mann–Whitney statistical test results, comparing the performance of six binarization experiments—Z4-STD, Z4-COM, Z4-ELIT, X4-STD, X4-COM, and X4–ELIT—across three algorithms: RSA, PSO, and GWO. This analysis spans both the Set Covering Problem and the 0–1 Knapsack Problem, offering insights into the effectiveness of these combinations in optimizing each problem.

In the SCP context, the RSA algorithm demonstrates the most consistent and statistically significant results across all binarization schemes. Specifically, the RSA achieves *p*-values ranging from 0.00 to 0.03, indicating its superior performance in identifying better solutions compared to other algorithms. Notably, the RSA’s adaptability is evident in its significant performance across both the Z4 and X4 transfer functions, with consistently low *p*-values for Z4-STD (p=0.00) and X4-ELIT (p=0.01). These results highlight the RSA’s ability to balance exploration and exploitation effectively, leading to robust optimization outcomes.

PSO also performs well in the SCP domain, particularly with binarization schemes such as Z4-ELIT (p=0.00) and X4-STD (p=0.03). However, it falls short in distinguishing performance for X4-COM (p=0.10), suggesting that this specific combination does not capitalize on PSO’s inherent strengths. The algorithm exhibits a broader range of *p*-values compared to the RSA, indicating some variability in its effectiveness depending on the choice of binarization scheme.

The GWO’s performance in the SCP is less consistent than the RSA but still exhibits notable strengths with schemes like Z4-STD (p=0.01) and Z4-ELIT (p=0.00). However, the non-significant results for Z4-COM (p=0.50) and X4-COM (p=0.10) suggest that the GWO struggles to leverage these schemes effectively. This variability underscores the GWO’s sensitivity to binarization schemes, where specific configurations significantly influence its optimization capability.

In the KP context, the RSA continues to outperform its counterparts with all *p*-values falling below 0.02. This consistency highlights the RSA’s adaptability to different problem structures and its capability to maintain strong performance regardless of the chosen binarization scheme. The significant results for Z4-STD (p=0.00) and X4-STD (p=0.00) demonstrate the RSA’s ability to harness the strengths of both Z4 and X4 transfer functions effectively.

The GWO also delivers significant results across all binarization schemes in the KP context, with *p*-values ranging from 0.01 to 0.03. This consistency across schemes and transfer functions reflects the GWO’s robustness in tackling KP instances. However, compared to RSA, GWO’s *p*-values are slightly higher, suggesting a marginally lower overall performance.

PSO exhibits mixed results in the KP context, with significant *p*-values for Z4-ELIT (p=0.00) and X4-ELIT (p=0.02), indicating its effectiveness in exploiting these schemes. However, non-significant results for Z4-COM (p=0.05) and X4-COM (p=0.05) suggest that PSO’s performance is less consistent and depends heavily on the specific binarization scheme employed.

Across both SCP and KP, Z4-ELIT and X4-ELIT emerge as the most reliable and effective binarization schemes. These schemes consistently deliver significant results across all three algorithms, highlighting their ability to enhance the algorithms’ optimization processes. On the other hand, Z4-COM and X4-COM frequently show non-significant differences, suggesting limited utility in distinguishing between algorithmic performances.

Finally, we can state that the RSA is the most versatile and effective algorithm across both problem contexts, consistently outperforming the GWO and PSO with statistically significant results for all binarization schemes. GWO demonstrates robustness but with slightly less consistent results compared to RSA, while PSO shows variability depending on the scheme and problem context. These findings emphasize the importance of aligning the choice of algorithm and binarization scheme to the specific problem characteristics to achieve optimal performance. The observed significant differences underscore the role of tailored configurations in leveraging problem-specific features, paving the way for superior optimization outcomes.

### 5.4. Summary of the Analysis

The analysis presented in this work reveals consistent patterns across the different evaluation dimensions, highlighting the critical role of binarization rules in determining algorithm performance, often outweighing the impact of the accompanying transfer function. While variations in performance are observed, certain trends emerge that provide valuable insights into the behavior of the algorithms and their configurations.

In the case of the SCP, the Elitist binarization rule consistently outperforms the other two across most instances and algorithms. While the Complement binarization rule occasionally demonstrates superior performance compared to the Standard rule, this behavior is not consistent across all statistical tests. Consequently, the Standard binarization rule emerges as the least effective choice for the SCP, failing to match the performance of the Elitist and Complement rules in most scenarios.

In contrast, the 0–1 Knapsack Problem reveals an inverse pattern. Here, the Standard binarization rule consistently delivers the best performance, while the Elitist rule is frequently the worst performer. The Complement rule, though occasionally surpassing the Elitist rule, does not consistently outperform the Standard rule. Among the algorithms, the GWO showcases three instances where the Complement rule achieves better results than the Elitist rule. Similarly, the PSO algorithm demonstrates a clear advantage only when using the Standard rule, with no consistent pattern of strong performance across the other binarization schemes.

The RSA demonstrates a distinct behavior compared to the GWO and PSO. The RSA delivers consistently strong results with both the Standard and Complement binarization rules, with the Standard rule being the best performer in the KP context. This finding aligns with the RSA’s demonstrated versatility across different optimization problems, suggesting that it maintains robust performance even with binarization rules that may be suboptimal for specific problems. This adaptability sets the RSA apart, reinforcing its suitability for a broader range of optimization tasks.

The statistical tests conducted provide further clarity on these patterns. While the tests do not measure raw performance directly, they highlight statistically significant differences between the performance of various binarization rule and transfer function combinations. The results underscore the critical role of binarization rules in driving algorithm performance, with the Elitist rule dominating SCP performance and the Standard rule leading in the KP context. However, examining the SCP results table reveals that the Z4 transfer function performs competently across a variety of binarization rules and algorithms. This consistency suggests that while binarization rules are the primary determinant of performance, the Z4 transfer function remains a reliable choice across the evaluated tests.

Summarizing, the analysis underscores the importance of selecting the appropriate binarization rule and transfer function for a given problem. The Elitist and Standard rules emerge as the strongest performers for SCP and KP, respectively, with the RSA demonstrating exceptional versatility across both problems. These findings emphasize the need for tailored algorithm configurations to optimize performance, providing a robust foundation for future work in algorithmic optimization.

## 6. Conclusions and Future Work

The objectives of this work, as outlined in the introduction, were successfully achieved. The Reptile Search Algorithm was rigorously evaluated on the Set Covering Problem and the 0–1 Knapsack Problem using various combinations of transfer functions and binarization rules. Its performance was compared against two established metaheuristics, Particle Swarm Optimization and the Grey Wolf Optimizer, providing valuable insights into the effectiveness of the RSA under diverse problem scenarios. Additionally, the binarization process was thoroughly analyzed, shedding light on its pivotal role in influencing algorithmic outcomes.

The RSA has demonstrated a consistently competitive performance against PSO and GWO, excelling across both tested problems. This versatility underscores its adaptability to different optimization contexts. In the SCP, the RSA particularly excelled in larger problem instances, where multiple combinations of transfer functions and binarization rules produced low RPD values. In the KP, the RSA consistently outperformed the other algorithms, achieving its best results when paired with the Z4-STD binarization experiment.

The convergence curves and exploration–exploitation results provide a detailed view of the RSA’s behavior, showcasing the flexibility afforded by its multiple movement equations. These equations allow the RSA to dynamically adapt its search strategy, with some equations proving more effective for specific problem characteristics. This adaptability underpins the RSA’s strong performance across both maximization and minimization problems.

The statistical analysis highlights the critical role of binarization rules in the binarization process. For SCP, the Elitist rule consistently delivered the best performance across all algorithms, regardless of the transfer function. For KP, the Standard rule emerged as the most effective, though RSA demonstrated strong results with the Complement rule as well. Notably, the Z4 transfer function mitigated performance losses when paired with less effective binarization rules, delivering competent results across all combinations. In contrast, the X4 transfer function showed significant performance degradation when not paired with the Elitist rule.

These findings emphasize the importance of tailoring algorithmic configurations to specific problem characteristics, aligning with the no-free-lunch theorem. The varying performances across transfer functions and binarization rules underscore the necessity of thoroughly understanding the problem at hand to select the most effective optimization strategy. While no universal algorithm guarantees optimal solutions across all scenarios, the RSA’s demonstrated flexibility and robustness make it a reliable choice for tackling a wide range of optimization problems.

The results also underscore the potential of the Z4 transfer function, which, while not universally optimal, significantly boosts performance with suboptimal binarization rules. This finding opens avenues for further research into the Z4 family and its potential to enhance two-step binarization methods for continuous metaheuristics applied to discrete problems.

Therefore, we can conclude that the RSA has proven to be a versatile and robust algorithm, capable of delivering strong performance across diverse problem domains. Its ability to adapt to both minimization and maximization problems makes it a valuable tool for addressing complex optimization challenges. Future work should focus on further exploring the RSA’s capabilities, particularly its flexibility and adaptability, while investigating the Z4 transfer function and its variants to improve the two-step binarization process and optimize results in discrete optimization problems. These insights lay a strong foundation for advancing metaheuristic design and application in complex problem-solving scenarios.

As future work, we propose to continue exploring the RSA’s performance across a broader range of optimization problems, including dynamic and large-scale scenarios, to further validate its flexibility and robustness. Additionally, an in-depth study of the Z4 transfer function and its family is suggested to enhance the effectiveness of the binarization process, particularly in cases where optimal binarization rules are not applied. Finally, the development of hybrid approaches that integrate RSA with other metaheuristics or machine learning techniques could open new avenues for tackling highly complex combinatorial optimization problems with improved precision and efficiency.

## Figures and Tables

**Table 1:** Transfer functions: S-shaped, V-shaped, X-shaped, and Z-shaped.

ID	Type	Equation	ID	Type	Equation
S1	S-shaped	$T\left(d_{w}^{j}\right)=\frac{1}{1+e^{-2d_{w}^{j}}}$	X1	X-shaped	$T\left(d_{w}^{j}\right)=\frac{1}{1+e^{2d_{w}^{j}}}$
S2	S-shaped	$T\left(d_{w}^{j}\right)=\frac{1}{1+e^{-d_{w}^{j}}}$	X2	X-shaped	$T\left(d_{w}^{j}\right)=\frac{1}{1+e^{d_{w}^{j}}}$
S3	S-shaped	$T\left(d_{w}^{j}\right)=\frac{1}{1+e^{-d_{w}^{j}/2}}$	X3	X-shaped	$T\left(d_{w}^{j}\right)=\frac{1}{1+e^{d_{w}^{j}/2}}$
S4	S-shaped	$T\left(d_{w}^{j}\right)=\frac{1}{1+e^{-d_{w}^{j}/3}}$	X4	X-shaped	$T\left(d_{w}^{j}\right)=\frac{1}{1+e^{d_{w}^{j}/3}}$
V1	V-shaped	$T\left(d_{w}^{j}\right)=\left|\mathrm{erf}\left(\sqrt{\pi }/2\cdot d_{w}^{j}\right)\right|$	Z1	Z-shaped	$T\left(d_{w}^{j}\right)=\sqrt{1-2^{d_{w}^{j}}}$
V2	V-shaped	$T\left(d_{w}^{j}\right)=\left|\tan h\left(d_{w}^{j}\right)\right|$	Z2	Z-shaped	$T\left(d_{w}^{j}\right)=\sqrt{1-5^{d_{w}^{j}}}$
V3	V-shaped	$T\left(d_{w}^{j}\right)=\left|\frac{d_{w}^{j}}{\sqrt{1+{\left(d_{w}^{j}\right)}^{2}}}\right|$	Z3	Z-shaped	$T\left(d_{w}^{j}\right)=\sqrt{1-8^{d_{w}^{j}}}$
V4	V-shaped	$T\left(d_{w}^{j}\right)=\left|\frac{2}{\pi }\arctan\left(\pi /2\cdot d_{w}^{j}\right)\right|$	Z4	Z-shaped	$T\left(d_{w}^{j}\right)=\sqrt{1-20^{d_{w}^{j}}}$

**Table 2:** Binarization rules.

Type	Binarization Rule
Standard	$X_{new}^{j}=\left\{\begin{array}{cc} 1, & \mathrm{if}\;rand\leq T(d_{w}^{j})\\ 0, & \mathrm{otherwise}. \end{array}\right.$
Complement	$X_{new}^{j}=\left\{\begin{array}{cc} \mathrm{Complement}(X_{w}^{j}), & \mathrm{if}\;rand\leq T(d_{w}^{j})\\ 0, & \mathrm{otherwise}. \end{array}\right.$
Static Probability	$X_{new}^{j}=\left\{\begin{array}{cc} 0, & T(d_{w}^{j})\leq \alpha \\ X_{w}^{j}, & \alpha <T\left(d_{w}^{j}\right)\leq \frac{1}{2}\left(1+\alpha \right)\\ 1, & T\left(d_{w}^{j}\right)>\frac{1}{2}\left(1+\alpha \right). \end{array}\right.$
Elitist	$X_{new}^{j}=\left\{\begin{array}{cc} X_{Best}^{j}, & rand<T(d_{w}^{j})\\ 0, & \mathrm{otherwise}. \end{array}\right.$
Roulette Elitist	$P\left[X_{new}^{j}=\zeta_{j}\right]=\frac{f(\zeta )}{\sum_{\delta \in Q_{g}}f\left(\delta \right)},\mathrm{if}\;rand\leq T\left(d_{w}^{j}\right)$

**Table 3:** Parameter settings of metaheuristics.

MH	Parameter	Value
Reptile Search Algorithm	α	0.1
	β	0.1
Particle Swarm Optimization	$w_{max}$	0.9
	$w_{min}$	0.1
	c1	2
	c2	2
Grey Wolf Optimizer	*a*	linearly decreased from 2 to 0

**Table 4:** Results obtained using RSA compared to PSO and GWO across SCP instances (scp41, scp51, and scp61).

Experiment	SCP41			SCP51			SCP61		
	Best	Avg.	RPD	Best	Avg.	RPD	Best	Avg.	RPD
RSA Z4-STD	434	433.07	1.17	270	268.65	6.72	142	141.07	2.90
RSA Z4-COM	460	452.39	7.23	277	274.84	9.49	145	143.07	5.07
RSA Z4-ELIT	433	433	0.93	269	268.45	6.32	141	141	2.17
RSA X4-STD	6820	5912.03	1489.74	13,250	12,230.26	5137.15	6537	6018.55	4636.96
RSA X4-COM	3656	3144.23	752.21	6821	6279.74	2596.05	3369	2851.19	2341.30
RSA X4-ELIT	434	433.61	1.17	269	267.77	6.32	141	141	2.17
PSO Z4-STD	455	447.52	6.06	278	273.55	9.88	151	147.87	9.42
PSO Z4-COM	463	463	7.93	289	289	14.23	159	159	15.22
PSO Z4-ELIT	442	437.90	3.03	274	270.55	8.30	145	142.77	5.07
PSO X4-STD	21,372	20,912.29	4881.82	44,583	43,686.07	17,521.74	21,493	20,849.26	15,474.64
PSO X4-COM	10,928	10,138.10	2447.32	22,148	21,317.81	8654.15	10,808	10,162.68	7731.88
PSO X4-ELIT	435	433.55	1.40	268	267.36	5.93	145	141.84	5.07
GWO Z4-STD	452	450.71	5.36	282	280.58	11.46	152	149.07	10.15
GWO Z4-COM	463	459.71	7.93	289	285.48	14.23	157	153.81	13.77
GWO Z4-ELIT	452	450.29	5.36	285	281.68	12.65	152	151.10	10.15
GWO X4-STD	20,137	19,674.13	4593.94	42,630	41,981	16,749.80	20,109	19,682.90	14,471.74
GWO X4-COM	10,834	10,101.29	2425.41	22,077	21,094.19	8626.09	10,598	10,201.97	7579.71
GWO X4-ELIT	438	434.19	2.10	269	266.52	6.32	146	141.77	5.80

**Table 5:** Results obtained using RSA compared to PSO and GWO across SCP instances (scpa1, scpb1, and scpc1).

Experiment	SCPA1			SCPB1			SCPC1		
	Best	Avg.	RPD	Best	Avg.	RPD	Best	Avg.	RPD
RSA Z4-STD	434	433.07	1.17	270	268.65	6.72	142	141.07	2.90
RSA Z4-COM	460	452.39	7.23	277	274.84	9.49	145	143.07	5.07
RSA Z4-ELIT	433	433	0.93	269	268.45	6.32	141	141	2.17
RSA X4-STD	6820	5912.03	1489.74	13,250	12,230.26	5137.15	6537	6018.55	4636.96
RSA X4-COM	3656	3144.23	752.21	6821	6279.74	2596.05	3369	2851.19	2341.30
RSA X4-ELIT	434	433.61	1.17	269	267.77	6.32	141	141	2.17
PSO Z4-STD	276	270.74	9.09	73	71.68	5.80	250	245.68	10.13
PSO Z4-COM	288	288	13.83	77	77	11.59	258	258	13.66
PSO Z4-ELIT	266	263.03	5.14	72	70.58	4.35	244	240.39	7.49
PSO X4-STD	21,372	20,912.29	4881.82	44,583	43,686.07	17,521.74	21,493	20,849.26	15,474.64
PSO X4-COM	10,928	10,138.10	2447.32	22,148	21,317.81	8654.15	10,808	10,162.68	7731.88
PSO X4-ELIT	435	433.55	1.40	268	267.36	5.93	145	141.84	5.07
GWO Z4-STD	452	450.71	5.36	282	280.58	11.46	152	149.07	10.15
GWO Z4-COM	463	459.71	7.93	289	285.48	14.23	157	153.81	13.77
GWO Z4-ELIT	452	450.29	5.36	285	281.68	12.65	152	151.10	10.15
GWO X4-STD	20,137	19,674.13	4593.94	42,630	41,981	16,749.80	20,109	19,682.90	14,471.74
GWO X4-COM	10,834	10,101.29	2425.41	22,077	21,094.19	8626.09	10,598	10,201.97	7579.71
GWO X4-ELIT	438	434.19	2.10	269	266.52	6.32	146	141.77	5.80

**Table 6:** Results obtained using RSA compared to PSO and GWO across SCP instances (scpd1, scpnre1, and scpnrf1).

Experiment	SCPD1			SCPNRE1			SCPNRF1		
	Best	Avg.	RPD	Best	Avg.	RPD	Best	Avg.	RPD
RSA Z4-STD	64	62.52	6.67	29	29	0	14	14	0
RSA Z4-COM	64	63.03	6.67	29	29	0	14	14	0
RSA Z4-ELIT	63	62.03	5	29	29	0	15	14.03	7.14
RSA X4-STD	27,176	25,181.90	45,193.33	33,555	31,938.16	115,606.90	32,891	31,206.87	234,835.71
RSA X4-COM	13,979	13,078.74	23,198.33	17,797	16,481.16	61,268.97	17,657	16,168.36	126,021.43
RSA X4-ELIT	64	61.90	6.67	29	29	0	14	14	0
PSO Z4-STD	65	64.29	8.33	30	29.71	3.45	15	14.45	7.14
PSO Z4-COM	74	74	23.33	30	30	3.45	16	16	14.29
PSO Z4-ELIT	64	62.74	6.67	29	29	0	14	14	0
PSO X4-STD	91,121	89,839.55	151,768.33	114,921	113,198.68	396,179.31	112,085	110,462.29	800,507.14
PSO X4-COM	45,380	43,966.52	75,533.33	57,325	55,648.03	197,572.41	55,529	53,737.07	396,535.71
PSO X4-ELIT	63	61	5	29	29	0	14	14	0
GWO Z4-STD	66	64.84	10	30	29.29	3.45	14	14	0
GWO Z4-COM	67	65.65	11.67	30	30	3.45	15	14.03	7.14
GWO Z4-ELIT	67	65.42	11.67	30	29.52	3.45	15	14.13	7.14
GWO X4-STD	88,034	87,296.07	146,623.33	111,264	110,359.45	383,568.97	108,420	107,521.81	774,328.57
GWO X4-COM	45,011	43,815.13	74,918.33	57,418	55,546.45	197,893.10	55,189	53,895.84	394,107.14
GWO X4-ELIT	63	61.29	5	30	29.03	3.45	14	14	0

**Table 7:** Results obtained using RSA compared to PSO and GWO across KP instances (knapPI_1_1000_1000_1, knapPI_1_2000_1000_1, knapPI_1_5000_1000_1, and knapPI_1_500_1000_1).

Experiment	knapPI_1_1000_1000_1			knapPI_1_2000_1000_1			knapPI_1_5000_1000_1			knapPI_1_500_1000_1		
	Best	Avg.	RPD	Best	Avg.	RPD	Best	Avg.	RPD	Best	Avg.	RPD
RSA Z4-STD	54,481	53,657.94	0.04	110,527	108,704.23	0.09	275,501	269,609.52	0.35	28,834	28,706.03	0.08
RSA Z4-COM	51,089	48,144.52	6.26	99,729	93,740.36	9.85	241,678	231,302.94	12.58	27,845	26,575.94	3.51
RSA Z4-ELIT	49,295	46,932.87	9.56	94,553	90,570.58	14.53	222,387	216,146.10	19.56	27,593	26,046.94	4.38
RSA X4-STD	50,998	49,778.65	6.43	97,453	95,654.77	11.91	229,638	226,318.03	16.94	28,522	27,732.77	1.16
RSA X4-COM	48,259	46,541.32	11.46	92,791	90,514.97	16.12	221,607	215,968.19	19.84	27,215	26,423.10	5.69
RSA X4-ELIT	48,755	46,556.16	10.55	93,817	90,542.13	15.19	223,432	216,584.23	19.18	27,438	25,965.19	4.92
PSO Z4-STD	49,289	46,587.61	9.57	96,401	90,523.23	12.86	221,078	216,113.32	20.03	27,196	25,973.26	5.76
PSO Z4-COM	49,755	46,608.77	8.71	93,351	91,143.81	15.62	221,600	216,779.29	19.84	27,625	26,010.61	4.27
PSO Z4-ELIT	49,720	46,711.13	8.78	95,563	90,726.45	13.62	220,902	215,472.71	20.10	27,572	25,997.29	4.45
PSO X4-STD	49,514	48,658.45	9.15	95,123	93,460.52	14.01	223,511	220,510.48	19.15	28,187	27,319.65	2.32
PSO X4-COM	48,188	46,470.65	11.59	94,790	90,434.32	14.31	225,099	216,503.10	18.58	26,974	25,877.36	6.53
PSO X4-ELIT	50,444	46,688.74	7.45	95,499	90,530.58	13.67	222,859	217,032.58	19.39	26,800	25,995.61	7.13
GWO Z4-STD	49,501	46,582.16	9.18	94,237	90,640.77	14.81	221,600	215,940.19	19.84	27,027	25,835.71	6.34
GWO Z4-COM	47,862	46,499.77	12.19	93,161	90,609.00	15.79	224,513	217,541.61	18.79	28,350	26,046.90	1.76
GWO Z4-ELIT	49,061	46,686.48	9.99	94,170	90,480.74	14.88	223,400	216,567.58	19.19	27,549	25,879.61	4.53
GWO X4-STD	50,012	49,055.19	8.24	95,487	93,643.84	13.68	222,202	219,401.39	19.63	28,109	27,597.84	2.59
GWO X4-COM	47,883	46,663.45	12.15	93,678	90,625.68	15.32	220,814	216,769.48	20.13	27,267	26,263.07	5.51
GWO X4-ELIT	49,638	46,967.90	8.93	94,693	90,163.94	14.40	223,660	216,917.07	19.10	26,793	25,962.61	7.15

**Table 8:** Results obtained using RSA compared to PSO and GWO across KP instances (knapPI_2_1000_1000_1, knapPI_2_2000_1000_1, knapPI_2_5000_1000_1, knapPI_2_500_1000_1).

Experiment	knapPI_2_1000_1000_1			knapPI_2_2000_1000_1			knapPI_2_5000_1000_1			knapPI_2_500_1000_1		
	Best	Avg.	RPD	Best	Avg.	RPD	Best	Avg.	RPD	Best	Avg.	RPD
RSA Z4-STD	9051	8971.03	0.01	18,003	17,878.19	0.27	44,281	43,858.90	0.17	4551	4524.68	0.33
RSA Z4-COM	8863	8646.16	2.09	17,396	17,015.16	3.63	42,216	41,502.55	4.83	4527	4420.48	0.85
RSA Z4-ELIT	8795	8608.13	2.84	16,991	16,718.97	5.87	40,855	40,503.71	7.89	4455	4408.48	2.43
RSA X4-STD	8900	8837.84	1.68	17,352	17,150.48	3.87	41,467	41,184.94	6.51	4551	4529.03	0.33
RSA X4-COM	8812	8642.81	2.65	16,992	16,717.61	5.87	40,875	40,507.58	7.85	4494	4441.74	1.58
RSA X4-ELIT	8736	8578.65	3.49	16,971	16,693.42	5.98	41,011	40,502.13	7.54	4496	4407.68	1.53
PSO Z4-STD	8764	8583.16	3.18	17,017	16,755.74	5.73	40,965	40,447.81	7.65	4502	4415	1.40
PSO Z4-COM	8784	8595.45	2.96	16,912	16,704.94	6.31	41,025	40,459.81	7.51	4489	4393.16	1.69
PSO Z4-ELIT	8748	8593.65	3.36	16,985	16,703.87	5.91	40,922	40,509.07	7.74	4501	4411.77	1.42
PSO X4-STD	8863	8749.68	2.09	17,217	16,983.07	4.62	41,061	40,815.48	7.43	4554	4507.77	0.26
PSO X4-COM	8698	8568.10	3.91	16,924	16,706.32	6.24	40,905	40,499.00	7.78	4488	4418.23	1.71
PSO X4-ELIT	8737	8587.48	3.48	16,925	16,717.61	6.24	41,206	40,584.90	7.10	4520	4410	1.01
GWO Z4-STD	8758	8593.52	3.25	17,024	16,720.65	5.69	40,845	40,483.97	7.92	4477	4403.94	1.95
GWO Z4-COM	8815	8614.19	2.62	17,064	16,771.65	5.47	40,945	40,520.39	7.69	4489	4404.36	1.69
GWO Z4-ELIT	8809	8599.03	2.68	17,013	16,721.87	5.75	41,142	40,556.26	7.25	4473	4390.61	2.04
GWO X4-STD	8874	8797.36	1.97	17,137	17,017.74	5.06	40,970	40,779.74	7.63	4552	4520.68	0.31
GWO X4-COM	8797	8600.13	2.82	16,994	16,758.26	5.86	40,913	40,468.65	7.76	4514	4454.16	1.14
GWO X4-ELIT	8828	8618.87	2.48	17,004	16,702.29	5.80	40,811	40,445.77	7.99	4487	4409.23	1.73

**Table 9:** Results obtained using RSA compared to PSO and GWO across KP instances (knapPI_3_1000_1000_1, knapPI_3_2000_1000_1, knapPI_3_5000_1000_1, knapPI_3_500_1000_1).

Experiment	knapPI_3_1000_1000_1			knapPI_3_2000_1000_1			knapPI_3_5000_1000_1			knapPI_3_500_1000_1		
	Best	Avg.	RPD	Best	Avg.	RPD	Best	Avg.	RPD	Best	Avg.	RPD
RSA Z4-STD	14,290	14,244.16	0.69	28,918	28,608.74	0.00	71,990	71,278.10	0.71	7117	7062.03	0.00
RSA Z4-COM	13,683	13,367.03	4.91	26,919	26,209.52	6.92	65,998	64,543.16	8.98	6915	6765	2.84
RSA Z4-ELIT	13,469	13,025.19	6.40	26,111	25,353.71	9.71	62,904	61,789.87	13.24	6916	6662	2.82
RSA X4-STD	13,675	13,499.97	4.97	26,416	26,063.94	8.66	63,998	63,216.26	11.73	7010	6911.74	1.50
RSA X4-COM	13,461	13,072.84	6.46	25,910	25,247.65	10.41	62,801	61,665.23	13.38	6816	6693.13	4.23
RSA X4-ELIT	13,275	13,023.07	7.75	26,014	25,265.87	10.05	62,598	61,664.90	13.66	6816	6656.77	4.23
PSO Z4-STD	13,284	13,025.94	7.69	25,809	25,263.16	10.75	62,604	61,887.68	13.66	6815	6696.39	4.24
PSO Z4-COM	13,381	13,012.10	7.01	25,618	25,208.48	11.42	62,802	61,780.90	13.38	6915	6698.58	2.84
PSO Z4-ELIT	13,379	13,029.65	7.03	25,719	25,270.81	11.07	63,004	61,752.48	13.10	6817	6673.52	4.22
PSO X4-STD	13,490	13,268.84	6.25	25,819	25,645.19	10.72	62,597	62,161.71	13.67	6917	6854.97	2.81
PSO X4-COM	13,287	12,986.03	7.67	25,818	25,368.39	10.72	62,698	61,667.71	13.53	6816	6654.87	4.23
PSO X4-ELIT	13,489	13,035.94	6.26	25,713	25,321.68	11.09	62,698	61,732.45	13.53	6815	6654.55	4.24
GWO Z4-STD	13,268	12,985.65	7.80	26,119	25,339.29	9.68	62,500	61,748.65	13.80	6906	6687.48	2.97
GWO Z4-COM	13,489	13,057.48	6.26	26,005	25,332.23	10.08	62,699	61,745.39	13.52	6807	6630.42	4.36
GWO Z4-ELIT	13,190	13,030.84	8.34	25,514	25,299.23	11.77	62,405	61,752.13	13.93	6815	6660.23	4.24
GWO X4-STD	13,589	13,346.94	5.57	26,219	25,707.61	9.34	63,001	62,091.07	13.11	7009	6867.16	1.52
GWO X4-COM	13,387	13,089.29	6.97	25,708	25,293.16	11.10	62,700	61,683.07	13.52	6866	6697.97	3.53
GWO X4-ELIT	13,282	13,025.32	7.70	25,618	25,313.77	11.42	62,397	61,714.10	13.94	6910	6658.81	2.91

**Table 10:** Summary of Wilcoxon–Mann–Whitney test results across all SCP and KP instances for each algorithm.

Algorithm	Z4-STD	Z4-COM	Z4-ELIT	X4-STD	X4-COM	X4-ELIT
Set Covering Problem						
Grey Wolf Optimizer	Significant (p=0.01)	Non-significant (p=0.50)	Significant (p=0.00)	Significant (p=0.05)	Non-significant (p=0.10)	Significant (p=0.02)
Particle Swarm Optimization	Significant (p=0.02)	Significant (p=0.05)	Significant (p=0.00)	Significant (p=0.03)	Non-significant (p=0.10)	Significant (p=0.02)
Reptile Search Algorithm	Significant (p=0.00)	Significant (p=0.03)	Significant (p=0.01)	Significant (p=0.00)	Significant (p=0.02)	Significant (p=0.01)
0–1 Knapsack Problem						
Grey Wolf Optimizer	Significant (p=0.01)	Significant (p=0.02)	Significant (p=0.01)	Significant (p=0.03)	Significant (p=0.02)	Significant (p=0.01)
Particle Swarm Optimization	Significant (p=0.01)	Non-significant (p=0.05)	Significant (p=0.00)	Significant (p=0.02)	Non-significant (p=0.05)	Significant (p=0.02)
Reptile Search Algorithm	Significant (p=0.00)	Significant (p=0.01)	Significant (p=0.01)	Significant (p=0.00)	Significant (p=0.02)	Significant (p=0.01)

## Data Availability

The original contributions presented in this study are included in the article. Further inquiries can be directed to the corresponding author.
